# The Occurrence and Characterization of Extended-Spectrum-Beta-Lactamase-Producing *Escherichia coli* Isolated from Clinical Diagnostic Specimens of Equine Origin

**DOI:** 10.3390/ani10010028

**Published:** 2019-12-21

**Authors:** Leta Elias, David C. Gillis, Tanya Gurrola-Rodriguez, Jeong Ho Jeon, Jung Hun Lee, Tae Yeong Kim, Sang Hee Lee, Sarah A. Murray, Naomi Ohta, Harvey Morgan Scott, Jing Wu, Artem S. Rogovskyy

**Affiliations:** 1 Department of Veterinary Pathobiology, College of Veterinary Medicine and Biomedical Sciences, Texas A&M University, College Station, TX 77845, USA; REevener@tamu.edu (L.E.); cgillis@cvm.tamu.edu (D.C.G.); tanya.gurrola@tamu.edu (T.G.-R.); smurray@cvm.tamu.edu (S.A.M.); hmscott@cvm.tamu.edu (H.M.S.); 2 National Leading Research Laboratory, Department of Biological Sciences, Myongji University, Yongin, Gyeonggido 17058, Korea; najashin@hanmail.net (J.H.J.); topmanlv@hanmail.net (J.H.L.); kty6523@gmail.com (T.Y.K.); sangheelee@mju.ac.kr (S.H.L.); 3 Faculty of Veterinary Medicine, Okayama University of Science, Imabari 794-8555, Japan; n-ohta@vet.ous.ac.jp; 4 Clinical Veterinary Microbiology Laboratory, College of Veterinary Medicine and Biomedical Sciences, Texas A&M University, College Station, TX 77845, USA; jwu@cvm.tamu.edu

**Keywords:** equine, ESBL, *Escherichia coli*, *Enterobacteriaceae*, antimicrobial resistance, CTX-M-1, SHV

## Abstract

**Simple Summary:**

The spread and development of extended spectrum beta-lactamase (ESBL)-mediated antimicrobial resistance is a significant concern in healthcare with impacts to animal and public health alike. While the occurrence of the ESBL phenotype in *Escherichia coli* has been investigated in depth by numerous studies, there is still a lack of information regarding ESBL-producing bacterial isolates from clinical specimens of equine origin. In this study, we investigated the incidence of ESBL-producing *E. coli* in hospitalized horses. Overall, 207 *E. coli* isolates were analyzed for their antimicrobial susceptibility and 13 ESBL-producing *E. coli* isolates were genotypically characterized. Seven out of the 13 *E. coli* isolates were found to harbor the resistance genes *bla*_CTX-M-1_ or *bla*_SHV-1_ and a novel beta-lactamase TEM gene variant, *bla*_TEM-233_ was discovered. Furthermore, despite being phenotypically susceptible to tested carbapenems, 1 out of 13 *E. coli* isolates was PCR-positive for the carbapenemase gene, *bla*_IMP-1_. The latter is an alarming finding because the presence of carbapenemase resistance genes in equine pathogens is extremely rare. In conclusion, equines can be reservoirs for ESBL-producing *Enterobacteriaceae*, and further investigation into this species group is necessary to understand their impact in the spread and development of antibiotic resistance genes.

**Abstract:**

*Escherichia coli* isolates were recovered from clinical specimens of equine patients admitted to the Texas A&M Veterinary Medical Teaching Hospital over a five-year period. Ceftiofur resistance was used as a marker for potential extended-spectrum beta-lactamase (ESBL)-activity, and of the 48 ceftiofur-resistant *E. coli* isolates, 27.08% (*n* = 13) were phenotypically ESBL-positive. Conventional PCR analysis followed by the _large-scale_*bla* Finder multiplex PCR detected the ESBL genes, CTX-M-1 and SHV, in seven out of the 13 isolates. Moreover, beta-lactamase genes of TEM-1-type, BER-type (AmpC), and OXA-type were also identified. Sequencing of these genes resulted in identification of a novel TEM-1-type gene, called *bla*_TEM-233_, and a study is currently underway to determine if this gene confers the ESBL phenotype. Furthermore, this report is the first to have found *E. coli* ST1308 in horses. This subtype, which has been reported in other herbivores, harbored the SHV-type ESBL gene. Finally, one out of 13 *E. coli* isolates was PCR-positive for the carbapenemase gene, *bla*_IMP-1_ despite the lack of phenotypically proven resistance to imipenem. With the identification of novel ESBL gene variant and the demonstrated expansion of *E. coli* sequence types in equine patients, this study underscores the need for more investigation of equines as reservoirs for ESBL-producing pathogens.

## 1. Introduction

In 2014, the World Health Organization (WHO) released a review that affirmed antimicrobial resistance as a major global threat, with a predicted impact of 100 trillion dollars in economic losses and 10 million deaths attributable to resistant bacteria by 2050 [1,2]. The production of beta-lactamases, a rapidly evolving class of hydrolytic enzymes that inactivate beta-lactam antibiotics, is a significant mechanism of antimicrobial resistance against penicillins and cephalosporins [3,4,5]. The wide application of beta-lactam antibiotics has been considered as a driving factor in the development and spread of extended-spectrum beta-lactamase (ESBL)-conferred resistance in Gram-negative bacterial pathogens such as *Escherichia coli, Klebsiella* spp., and *Salmonella* [4,5,6,7]. The ESBL-encoding genes (e.g., *bla*_CTX-M_, *bla*_SHV_) allow these pathogens to produce enzymes that hydrolyze the beta-lactam ring of penicillins, first-, second-, and third-generation cephalosporins, and aztreonam, although ESBL-positive pathogens still remain susceptible to carbapenems and cephamycins [8,9]. To date, ESBL-positive bacteria are rapidly emerging in a variety of host species worldwide and pose a serious threat to public health [1,4,10,11].

Analogous with human medicine, ESBL-production is a pronounced concern in the veterinary field [12,13,14,15]. Specifically, ESBL-producing *E. coli* have been reported as a cause of severe infections in horses [14,16]. Moreover, nosocomial transmission of ESBL-positive pathogens of the *Enterobacteriaceae* family between horses has also been discussed [13,14]. Of additional importance, the possibility of cross-species transmission of ESBL-positive bacterial strains directly represents a health hazard for humans, especially equine handlers and veterinary staff [15,17,18]. Despite this significant problem, however, information on the occurrence and genetic characterization of ESBL-positive *E. coli* isolated from horses is lacking [3,19,20]. Of the studies that have investigated ESBL-positive *E. coli* in equines, the isolates were predominantly originated from fecal samples. To date, very few studies have thoroughly evaluated the occurrence of ESBL-positive *E. coli* in diagnostic specimens (other than feces) from equine patients [14,19]. As such, the objective of the present work was to examine the proportion and genetic diversity of ESBL-positive *E. coli* in clinical specimens of equine origin.

## 2. Materials and Methods

### 2.1. Sample Collection and Bacterial Identification

A total of 207 *E. coli* isolates were recovered from equine clinical diagnostic specimens submitted to the Texas A&M Veterinary Medical Teaching Hospital (VMTH) between January 1, 2009 and December 31, 2014. Diagnostic specimens fell into the following categories: abscess (e.g., pus, draining tract swabs), abdominal cavity (e.g., peritoneal fluid), blood, colon, ear, liver, spleen, female and male reproductive systems (e.g., cervix, clitoral sinus, uterus, semen, prepuce), lower and upper respiratory tract (e.g., guttural pouch, transtracheal wash, lung), skeletal system (bone/bony sequestrum, hoof, and joint), surgical site (e.g., incision swab, screw), thoracic cavity (e.g., pleural fluid), urinary system (e.g., bladder, urine), and wound. 

All samples were processed immediately after they were submitted to the VMTH Clinical Veterinary Microbiology Laboratory (CVML). The isolates were identified as *E. coli* based on Gram stain, colony morphology, and biochemical analyses that included triple sugar agar, lysine iron agar, motility agar, citrate, indole, and urease tests [21]. Where identification was still in question, the RapID^TM^ One System (Remel, Lenexa, KS, USA) was utilized.

### 2.2. Antimicrobial Susceptibility Testing

*E. coli* isolates were tested for antimicrobial susceptibility via broth microdilution using commercially available TREK Sensititre™ Systems (Trek Diagnostic Systems, Inc, Oakwood Village, OH, USA). Since the clinical isolates were tested for their susceptibility against various antimicrobial classes as part of veterinary diagnostic service, and that this service was provided over the five-year period, minimum inhibitory concentration (MIC) data were derived from different antimicrobial susceptibility panels and hence the numbers of isolates tested per panel varied. In addition to Sensititre™ COMEQ3F Plate and Sensititre™ Equine EQUIN1F AST Plate (Trek Diagnostic Systems, Thermo Fisher Scientific, Lenexa, KS, USA), some isolates were tested by a newer panel, Sensititre™ NARMS Gram Negative Plate (Trek Diagnostic Systems, Thermo Fisher Scientific, Lenexa, KS, USA). Breakpoints from the most current Clinical and Laboratory Standards Institute (CLSI) guideline M100 were applied to interpret the MIC results [22]. The *E. coli* isolates that were resistant to ceftiofur were also tested using TREK Sensititre™ ESBL Plate (Trek Diagnostic Systems, Thermo Fisher Scientific, Lenexa, KS, USA). The confirmatory testing included both cefotaxime and ceftazidime. *E. coli* isolates were considered ESBL-positive if there was a 3 or greater two-fold concentration decrease in the MIC for cefotaxime or ceftazidime with clavulanic acid as compared to the MIC for the respective antimicrobial agent when tested alone. *E. coli* (ATCC^®^ 25922™) obtained from the American Type Culture Collection (Old Town Manassas, VA, USA) was used as a CLSI control strain.

### 2.3. Detection and Characterization of Bla Genes

Genomic DNA was isolated from ESBL-positive *E. coli* using QIAprep Spin™ Miniprep kit (Qiagen, Valencia, CA, USA) according to the manufacturer’s instruction. Beta-lactamase genes, *bla*_SHV_, *bla*_TEM_, and *bla*_CTX-M_ of groups 1, 2, 8, 9, and 10 were then screened by PCR as previously described [23,24]. The primer sequences are provided in Table S1. The amplicons of expected sizes were further sequenced at Molecular Cloning Laboratories (San Francisco, CA, USA) and the sequence results were verified via BLASTn [25]. 

In addition, a recently developed detection method, _large-scale_*bla* Finder (_large-scale_*bla* Finder, Dr. ProLab, Inc., Yongin, South Korea) was also utilized [26]. Specifically, a colony of a fresh overnight culture from LB medium plate was inoculated in 20 µL 0.1% Triton X-100 and then heated at 100 °C for 10 min. After centrifugation at 18,000× *g* for 1 min, the supernatant was used as a DNA template for the multiplex PCR. The multiplex mixture containing 1X Solg^TM^ Multiplex PCR Smart mix and 1 Unit of Uracil-DNA glycosylate (SolGent Co., Ltd., Daejeon, South Korea) was mixed with RNase-free water and primer mixture [26]. The final concentration of each primer was 0.2143 μM. Template DNA was then added to the mixture. Amplification was performed under the following thermal cycling conditions: initial denaturation at 95 °C for 5 min; 30 cycles of 95 °C for 30 s, 64 °C for 40 s, and 72 °C for 50 s; and a final elongation step at 72 °C for 7 min. After amplification samples were stored at 4 °C until further analysis. Resultant amplicons were analyzed by electrophoresis on a 2% agarose gel at 100 V for 1 h and with ethidium bromide staining and then sequenced (Molecular Cloning Laboratories, San Francisco, CA, USA).

### 2.4. Multi-Locus Sequence Typing (MLST)

To determine the sequence type (ST) of each isolate, seven housekeeping genes, *adk*, *fumC*, *gyrB*, *icd*, *mdh*, *purA*, and *recA* were PCR amplified as described [27]. Specifically, amplification was performed under the following conditions: initial denaturation at 95 °C for 2 min; 30 cycles of 95 °C for 2 min, 52 °C for 1 min, and 72 °C for 2 min; and a final elongation step at 72 °C for 5 min. Resulting sequenced amplicons were used to determine bacterial STs by using the *E. coli* MLST Database [28]. All the primer sequences are provided in Table S2.

### 2.5. *E. coli* Phylogroup Identification

*E. coli* phylogroup identification of ESBL-positive isolates was performed as described [29] and PCR conditions were as follows: initial denaturation at 95 °C for 5 min; 30 cycles of 95 °C for 30 s, 55 °C for 30 s, and 72 °C for 30 s; and a final elongation step at 72 °C for 7 min. All the primers are provided in Table S3.

### 2.6. Ethics

In the present study, all the bacterial isolates that were phenotypically and genotypically characterized had been recovered from clinical specimens of equine origin submitted to the VMTH. Since the clinical specimens were analyzed as part of veterinary diagnostic service, no specific approval on the animal subject was required for phenotypical or genotypical characterization of *E. coli* isolates.

## 3. Results

### 3.1. Phenotypic Analysis

Antimicrobial susceptibility testing detected resistance to ampicillin in 28.07% of the *E. coli* isolates (48/171; the number of resistant isolates/the total number of isolates tested; Table 1). Among the third-generation cephalosporins, the lowest proportion of resistance was observed for the *E. coli* isolates tested against ceftazidime, with only 3.25% (4/123) of the isolates being resistant to this antimicrobial. In contrast, when tested against ceftriaxone and ceftiofur, the numbers of resistant isolates were significantly higher, 29.17% (14/48) and 27.08% (13/48), respectively. Resistance to cefpodoxime was observed in 13.89% of the isolates tested (5/36). Lastly, resistance to cefoxitin, a second-generation cephalosporin, was only detected in 4.76% of the *E. coli* isolates (4/84; Table 1).

Resistance against chloramphenicol was identified in 21.64% of the equine isolates (37/171). Of note, 100% of the *E. coli* isolates were susceptible to amikacin (0/36), whereas 37.50% (18/48) and 20.83% (10/48) of the isolates were resistant to gentamicin and streptomycin, respectively (Table 1). Resistance to tetracycline was detected in 50.0% of the *E. coli* isolates (24/48), which was approximately twice as high when compared to resistance of the isolates against doxycycline (26.02%; 32/123). Resistance to ciprofloxacin and enrofloxacin was found in 29.17% (14/48) and 13.89% (5/36) of the equine isolates, respectively. Against nalidixic acid, 29.17% of the isolates (14/48) were resistant. Importantly, none of the isolates (0/159) were resistant to imipenem, the carbapenem antimicrobial, which is commonly used as a reserve drug to treat serious infections by ESBL-positive pathogens in humans [30]. When the isolates were tested against trimethoprim-sulfamethoxazole, resistance was observed in 37.43% of the isolates (64/171; Table 1). It is important to note that while the 207 isolates were tested for their antimicrobial susceptibility in total, some drugs that had breakpoints outside of the range of the susceptibility panels were excluded from the analysis despite their respective MIC data are still presented in Table 1.

Resistance to ceftiofur, which was detected in 13 out of 48 *E. coli* isolates tested, was an indicator of potential ESBL activity and prompted further susceptibility testing to confirm the ESBL phenotype (Table 1). All 13 ceftiofur-resistant isolates displayed the ESBL phenotype: three or greater two-fold concentration decrease in an MIC for cefotaxime or ceftazidime in combination with clavulanic acid compared to the MIC of the respective antimicrobial when tested alone [18,26]. Moreover, 46.15% (6/13), 76.92% (10/13), and 84.62% (11/13) of the isolates were resistant to cefepime, ceftriaxone, and cefpodoxime, respectively (Table 2). Testing against cefoxitin showed that two of the 13 isolates were resistant to this cephamycin. This finding suggested that these two isolates also harbored a cephamycinase gene that would confer resistance to cefoxitin while sustaining the ESBL phenotype–susceptibility in the presence of clavulanic acid to the third-generation cephalosporins. Consistently, all of the ESBL-positive isolates exhibited full susceptibility to imipenem and meropenem (Table 2).

### 3.2. Genetic Characterization of ESBL-Positive *E. coli* Isolates

In order to detect ESBL and other beta-lactamase genes in the 13 ESBL-positive *E. coli* isolates, in addition to conventional PCR [23,24], a more comprehensive multiplex PCR-based detection method, _large-scale_*bla* Finder [26] was used in the present study. Moreover, multilocus sequence typing (MLST) analysis was also utilized to determine sequence types of the 13 *E. coli* isolates. As a result, a total of six distinct *bla* gene types were identified: TEM, BER, SHV, CTX-M-1, OXA-1, and IMP (Table 3). Of these, the AmpC beta-lactamase gene, *bla*_BER_, was most represented among the isolates, with its proportion being 84.62% (11/13). A variant of previously characterized *bla*_BER_ gene (GenBank accession number: EF125541) was detected in one of the 13 isolates. This BER-type *bla* variant had five silent mutations, T375C, G378A, C387T, T477G, and T576A. Moreover, additional BER-type mutations were observed in *bla*_BER_ of another *E. coli* isolate. In addition to the five silent mutations of the original BER variant (GenBank accession number: EF125541), this BER variant 1 (BER-v1) had two additional nucleotide substitutions, G469A and G1012A, which translated into their respective amino-acid substitutions, A157T and G338S, in BER (GenBank accession number: ABM69263). Also, the study detected another BER-type variant (BER-v2), whose mutations were also consistent with the 5 silent mutations of the original BER variant (GenBank accession number: EF125541; Table 3). Yet, this BER-v2 had also four additional nucleotide substitutions, G245A, G313A, G469A, and G1012A (GenBank accession number: EF125541), which, respectively, translated into 4 amino-acid substitutions, S28N, A105T, A157T, and G338S (GenBank accession number: ABM69263). The second most commonly identified *bla* gene was TEM-1, which was found in 69.23% (9/13) of the *E. coli* isolates. Two silent mutations, C228T and G396T, in TEM-1 were consistently found in the nine isolates. CTX-M-1 was detected in 30.77% (4/13) of the *E. coli* isolates. Furthermore, SHV-12 and OXA-1 were detected in 23.07% (3/13) of the isolates each. Alarmingly, the metallo-beta-lactamase IMP-1 was detected in one out of the 13 isolates (Table 3). Lastly, a novel TEM-1-type beta-lactamase gene, designated as *bla*_TEM-233_, was detected in isolate E9A52022 (GenBank accession number: MH270416; Table 3).

### 3.3. Phylogenetic Grouping

The 13 ESBL-positive *E. coli* isolates belonged to four phylogenetic groups that were represented by a total of seven distinct sequence types (Table 3). Four isolates of ST10 (*n* = 2) and ST410 (*n* = 2) belonged to phylogroup A. Phylogroup B1 was most diverse and included four equine isolates with distinct sequence types: ST167, ST1308, ST224, and ST156. Phylogroup B2 had only two clinical isolates, ST168 and ST410. Lastly, phylogroup D was uniformly represented by three ST648 isolates (Table 3).

## 4. Discussion

In the present study, a total of 207 *E. coli* isolates were cultured from clinical diagnostic specimens collected from equine patients, which were admitted to the Texas A&M Veterinary Medical Teaching Hospital from 2009 through 2014. The results of the present study demonstrated that 27.08% of the isolates screened with ceftiofur expressed the ESBL-positive phenotype. Recent studies have reported the occurrence of ESBL-producing *E. coli* isolates recovered from equines to range from as low as 0.2% (one out of 508 isolates tested) in feral horses living on an isolated Canadian island [31], to 84% in equine patients at a veterinary teaching hospital in the Netherlands [19]. Additional studies detected ESBL-producing *E. coli* in 6.3% of fecal samples from equine patients across various veterinary practices in the United Kingdom [12], 10.1% of equine patients at a veterinary clinic in Germany [20], and 32% of equine patients at a veterinary clinic in the Czech Republic [3].

Resistance to chloramphenicol was present in a high proportion of the *E. coli* isolates (21.64%; 37/171). Due to its negative side effects, chloramphenicol is banned in human medicine and is considered a last choice drug to treat gastrointestinal disease (e.g., abdominal abscesses and salmonellosis) in horses [32]. Thus, the usage of chloramphenicol in equine medicine may explain the high proportion of resistance detected in the tested isolates.

Overall, the 13 ESBL-positive *E. coli* isolates represented a total of seven sequence types: ST648, ST410, ST10, ST224, ST167, ST1308, and ST156. Of these, ST648, ST167, ST410, ST224, and ST10 have been described as extended host spectrum genotypes [13]. ST648 was the most prevalent sequence type isolated in this study and encompassed a total of four ESBL-positive *E. coli* isolates that were recovered from three equine patients. *E. coli* ST648 is associated multi-drug resistance and high virulence, drawing comparisons with ST131, which is recognized as an internationally relevant high-risk *E. coli* [13,33]. *E. coli* ST648 was recovered from a variety of animals including canines, felines, horses, livestock, wild birds, and humans [34,35,36]. ST410, which was identified in 3 of the 13 *E. coli* isolates, has also been described as an emerging high-risk *E. coli* with potential international implications [37]. ST410 was previously isolated from humans [38,39], canines, felines [40], swine, poultry, cattle [41,42,43], as well as birds [44]. *E. coli* ST10, identified in two of the 13 isolates, was recovered from humans, turkey meat, chickens, swine, cattle [45,46], and horses [19,20]. Lastly, ST224, ST167, ST1308, and ST156 were detected in one isolate each. Notably, both ST10 and ST224 previously demonstrated their capacity for nosocomial infections and the spread of these sequence types between horses and potentially, to their human handlers [14,18,19]. In addition to being recovered from horses [14], ST224 was isolated from humans [11], swine [42], bovines [47], birds [44], and in this study, from a donkey. ST167 *E. coli* was isolated from humans, cattle, swine, wild birds [34,46,48], turkey meat [43], and horses [49]. Furthermore, this sequence type has been associated with the global carriage of ESBL-producing *E. coli* [13,34,48]. *E. coli* ST156 was identified in fish [50], canines, felines, horses [51], chickens, and other avian species [52,53]. Lastly, this study is the first demonstration of ST1380 *E. coli* being isolated from equines, the sequence type that was previously isolated from swine and bovine species [54,55].

Phylogenetic analysis showed that the 13 ESBL-positive *E. coli* isolates belonged to four phylogroups: A, B1, B2, and D. The most represented phylogroups were A and B1, with each encompassing 4 of the 13 ESBL-positive *E. coli* isolates. The phylogroup A, which commonly includes commensal strains of *E. coli* [29], was composed of the ST410 and ST10 isolates. The sequence types, ST224, ST167, ST1308, and ST156 belonged to phylogroup B1. Furthermore, three of the 4 ESBL-positive *E. coli* ST648 isolates fell into phylogroup group D. One of the ESBL-positive *E. coli* ST648 isolates belonged to phylogroup B2, which, in addition to phylogroup D, is most often associated with virulent extraintestinal infections [56]. Finally, phylogroup B2 included one ESBL-positive *E. coli* isolate with the sequence type ST410 [57]. It should be emphasized that despite the fact that phylogroups A and B1 are associated with commensal *E. coli*, which are considered harmless, these organisms can act as reservoirs for ESBL gene-carrying plasmids and, therefore, may contribute to the spread of resistance among pathogenic bacteria [57,58,59]. This, in turn, puts both humans and animals at risk for the nosocomial spread and cross-species transfer of ESBL resistance genes [13,15,17,18].

The 13 ESBL-positive *E. coli* isolates were screened for *bla*_SHV_, *bla*_TEM_, and *bla*_CTX_ of groups 1, 2, 8, 9, and 10. In addition to the conventional PCR-based approach [23,24], the recently developed _large-scale_*bla* Finder detection method was utilized to more thoroughly examine the isolates for the presence of most clinically relevant beta-lactamase genes [26]. As a result, a novel TEM-1-type beta-lactamase gene, designated as *bla*_TEM-233_, was detected in one *E. coli* isolate. Further investigation is needed to determine whether or not this newly identified variant is functional. Moreover, the TEM-1-type *bla* gene with two silent mutations at C228T and G396T was consistently detected in eight out of the 13 ESBL-positive isolates of the following sequence types: ST648, ST167, ST1308, ST224, and ST410. Of these TEM-1 harboring isolates, the most represented phylotype was B1, which included three of the 8 isolates. One *E. coli* isolate represented group B2 and the other four isolates belonged to groups A and D (two in each group). Furthermore, a variant of the AmpC beta-lactamase producing gene [60] was detected in one of the 13 isolates. This BER-type *bla* variant fell within phylogroup B1 and belonged to *E. coli* of ST1308. Additionally, BER-v1 (BER variant 1) was observed in one isolate of phylogroup D and ST648. Lastly, BER-v2 was of ST410 and belonged to phylogroup B2.

It should be noted that despite all 13 isolates that exhibited the ESBL phenotype, only seven isolates were genotypically confirmed to harbor ESBL-resistance genes. While the _large-scale_*bla* Finder method identifies a much wider array of clinically relevant *bla* genes compared to the conventional PCR approach, it does not detect all the existing ESBL genes. As such, it is well possible that some ESBL genes remained undetected in the other six isolates. Additionally, the carbapenemase gene, *bla*_IMP-1_, was found in isolate E7A44050DS despite the lack of detectable carbapenem resistance when tested phenotypically. The latter was a surprising finding because this carbapenemase gene had no mutations, which was determined through three independent sequencing runs. Previously, it was shown that MICs of carbapenem-producing *Enterobacteriaceae* may vary greatly and even be below the CLSI-established carbapenem breakpoints [61]. Moreover, two out of the seven genotypically-confirmed ESBL-positive isolates were also resistant to cefoxitin, a second-generation cephalosporin, which is not typical of ESBL-producing *E. coli* and is more commonly associated with AmpC beta-lactamase-producing bacteria [8,26,62]. Together, these interesting results warrant further genetic testing (e.g., via whole genome sequencing) for a more thorough analysis of these *E. coli* isolates.

## 5. Conclusions

In summary, this study demonstrated the first occurrence of *E. coli* ST1380 recovered from clinical specimens of equine origin, a finding that indicates a wider host-range for this *E. coli* ST than was previously reported. This ST1308 *E. coli* isolate harbored the *bla*_SHV-12_ ESBL gene, highlighting the necessity of studying the spread and development of ESBL genes in equines. Alarmingly, one *E. coli* isolate was PCR-positive for the carbapenemase gene, *bla*_IMP-1_ despite this isolate was phenotypically susceptible to imipenem. Lastly, as a result of genetic characterization of beta-lactamase-positive equine isolates, a novel TEM-1-like gene was identified and a study is currently underway to test if this novel ESBL gene is fully functional.

## Figures and Tables

**Table 1:** Distribution of minimum inhibitory concentrations of clinical *Escherichia coli* isolates of equine origin.

Antimicrobials	Isolates Tested	# Resistant Isolates ^a^	% Resistant Isolates	95% CI Lower	95% CI Upper	<0.015	0.015	0.03	0.06	0.125	0.25	0.5	1	2	4	8	16	32	64	128	256	512	1028
Amikacin ^b^	123	^													90.24	6.5	0	1.63	1.63				
Amikacin ^d^	36	0	0.00	0	9.74 *										86.11	5.56	8.33	0					
Amoxacillin/ Clavulanic Acid ^c^	48	4	8.33	2.32	19.98									4.17	16.67	31.25	27.08	6.25	2.08				
Amoxacillin/ Clavulanic Acid ^d^	36	3	8.33	1.75	22.47									0	38.89	52.78	0	5.56	2.78				
Ampicillin ^b^	123	27	21.95	14.99	30.31								5.69	33.33	35.77	2.44	0.81	0.81	21.14				
Ampicillin ^c^	48	21	43.75	29.48	58.82								8.33	22.92	20.83	4.17	0	0	43.75				
Ampicillin ^d^	36	^											5.56	19.44	13.89	0	0	61.11					
Azithromycin ^b^	123	^											4.07	15.45	50.41	30.08							
Azithromycin ^c^	48	7	14.58	6.07	27.76								4.17	12.5	52.08	14.58	2.08	14.58					
Cefazolin ^‡b^	123	^													91.87	2.44	0	5.69					
Cefazolin ^‡d^	36	^													0	80.56	8.33	11.11					
Cefoxitin ^c^	48	4	8.33	2.32	19.98									22.92	47.92	14.58	6.25	0	8.33				
Cefoxitin ^d^	36	0	0.00	0	9.74 *									38.89	41.67	11.11	8.33						
Cefpodoxime ^d^	36	5	13.89	4.67	29.50									80.56	5.56	2.78	11.11						
Ceftazodime ^b^	123	4	3.25	0.89	8.12								95.93	0.81	0	0	1.63	0	0	1.63			
Ceftiofur ^b^	123	^									44.72	48.78	0.81	0.81	0.81	4.07							
Ceftiofur ^c^	48	13	27.08	15.28	41.85					2.08	18.75	35.42	10.42	4.17	2.08	0	27.08						
Ceftiofur ^d^	36	^									50	27.78	5.56	5.56	2.78	8.33							
Ceftriaxone ^c^	48	14	29.17	16.95	44.06						62.5	2.08	2.08	4.17	2.08	0	2.08	6.25	2.08	16.67			
Cephalothin ^d^	36	^													13.89	27.78	36.11	22.22					
Chloramphenicol ^b^	123	23	18.70	12.24	26.72										37.4	37.4	6.5	2.44	16.26				
Chloramphenicol ^c^	48	14	29.17	16.95	44.06									6.25	22.92	33.33	8.33	2.08	27.08				
Chloramphenicol ^d^	36	^													36.11	38.89	0	25					
Ciprofloxacin ^c^	48	14	29.17	16.95	44.06		64.58	2.08	0	0	4.17	0	0	0	29.17								
Doxycycline ^b^	123	32	26.02	18.52	34.70									65.04	7.32	1.63	8.13	17.89					
Enrofloxacin ^b^	123	^									88.62	0.81	1.63	0	8.94								
Enrofloxacin ^d^	36	5	13.89	4.67	29.50							86.11	0	0	0	13.89							
Gentamicin ^b^	123	^											69.92	6.5	1.63	0.81	21.14						
Gentamicin ^c^	48	18	37.50	23.95	52.65							10.42	39.58	10.42	0	2.08	0	37.5					
Gentamicin ^d^	36	^											44.44	8.33	0	0	47.22						
Imipenem ^b^	123	0	0.00	0.00	2.95*								100	0	0								
Imipenem^d^	36	0	0.00	0.00	9.74*								100	0	0								
Marbofloxacin ^d^	36	^									86.11	2.78	0	0	11.11								
Naladixic Acid ^c^	48	14	29.17	16.95	44.06								12.5	39.58	14.58	4.17	0	0	29.17				
Orbifloxacin ^d^	36	^											86.11	0	2.78	11.11							
Streptomycin ^c^	48	10	20.83	10.47	34.99										4.17	52.08	16.67	6.25	0	20.83			
Sulfisoxazole ^c^	48	^															50	8.33	0	2.08	0	39.58	
Tetracycline ^b^	123	^												69.92	1.63	0.81	27.64						
Tetracycline ^c^	48	24	50.00	35.23	64.77										50	0	0	4.17	45.83				
Tetracycline ^d^	36	^												47.22	0	0	52.78						
Ticarcillin ^b^	123	^														77.24	0	0	0.81	21.95			
Ticarcillin ^d^	36	^														36.11	2.78	5.56	2.78	52.78			
Ticarcillin/ Clavulanic acid ^b^	123	^														88.62	4.07	3.25	0.81	3.25			
Ticarcillin/ Clavulanic acid ^d^	36	0	0.00	0.00	9.74*											72.22	16.67	8.33	2.78				
Trimethoprim-Sulfamethoxazole ^b^	123	45	36.59	28.09	45.75								62.6	0.81	0	36.59							
Trimethoprim-Sulfamethoxazole ^c^	48	19	39.58	25.77	54.73					60.42	0	0	0	0	0	39.58							
Trimethoprim-Sulfamethoxazole ^d^	36	^										33.33	5.56	0	61.11								

Resistance profiles of 207 *E. coli* isolates from equine patients of Texas A&M University Teaching Hospital; ^a^ The interpretation of minimum inhibitory concentration (MIC) was based on the 2019 Clinical and Laboratory Standards Institute (CLSI) guideline M100 (indicated by vertical red bars) unless otherwise specified; ^b^ Sensititre™ Equine EQUIN1F AST Plate; ^c^ Sensititre™ NARMS Gram Negative Plate; ^d^ Sensititre™ COMEQ3F Plate; * one-sided, 97.5% confidence interval; ^ CLSI MIC breakpoint is above the range of the assay; ^‡^ CLSI breakpoints for oral cefazolin were used to interpret the MIC.

**Table 2:** Antimicrobial susceptibility of Extended-Spectrum Beta-Lactamase (ESBL)-positive *Escherichia coli* isolates of equine origin.

Antimicrobial	MIC (μg/mL)												
	E1A	E2A	E3A	E4A	E4B	E4C	E5A	E6A	E7ARL	E7ADS	E8A	E8B	E9A
Cefazolin ^a^	>16	>16	16	>16	16	>16	>16	>16	>16	>16	>16	>16	>16
Cefepime	16	2	≤1	16	≤1	>16	16	8	2	4	16	>16	≤1
Cefotaxime	64	16	0.5	>64	1	>64	>64	>64	8	16	>64	>64	1
Cefotaxime/Clavulanic acid	≤0.12	≤0.12	≤0.12	≤0.12	≤0.12	0.25	≤0.12	≤0.12	≤0.12	≤0.12	≤0.12	8	≤0.12
Cefoxitin	16	≤4	≤4	16	≤4	32	8	8	≤4	≤4	8	>64	8
Cefpodoxime	>32	>32	8	>32	4	>32	>32	>32	>32	>32	>32	>32	4
Ceftazidime	8	4	4	16	16	16	64	0.5	2	2	16	64	16
Ceftazidime/Clavulanic acid	0.25	≤0.12	≤0.12	0.25	≤0.12	0.5	0.5	≤0.12	0.50	0.25	0.25	16	≤0.12
Ceftriaxone	128	32	≤1	128	≤1	>128	>128	32	64	32	128	128	2
Cephalothin	>16	>16	>16	>16	>16	>16	>16	>16	>16	>16	>16	>16	>16
Ciprofloxacin	>2	>2	≤1	>2	>2	>2	>2	>2	≤1	≤1	>2	>2	>2
Imipenem	≤0.5	≤0.5	≤0.5	≤0.5	≤0.5	≤0.5	≤0.5	≤0.5	≤0.5	≤0.5	≤0.5	≤0.5	≤0.5
Meropenem	≤1	≤1	≤1	≤1	≤1	≤1	≤1	≤1	≤1	≤1	≤1	≤1	≤ 1
Piperacillin-Tazobactam	8	≤4	≤4	16	≤4	16	16	≤ 4	≤4	≤4	16	16	≤ 4

^a^ The CLSI breakpoint for oral cefazolin was used to interpret the MIC.

**Table 3:** The *bla* genes detected in ESBL-positive *Escherichia coli* isolates of equine origin.

Isolate ID	*bla* Gene Type Detected Using _large-scale_*bla* Finder Kit	*bla* Gene Name by Sequencing of Simplex PCR Products Using Long-Length Primer Pairs of _large-scale_*bla* Finder Kit to Detect Each ORF (GenBank Accession No. of Gene)	Phylogroup	MLST
E1A17025	TEM type	Two silent mutations (C228T and G396T) in *bla*_TEM-1_ (J01749)	D	648
E2A28099DS	TEM type	Two silent mutations (C228T and G396T) in *bla*_TEM-1_ (J01749)	B1	167
	BER type	*bla*_BER_^a^ (EF125541)		
	BER type	*bla*_BER_^a^ (EF125541)		
E3A31074	TEM type	Two silent mutations (C228T and G396T) in *bla*_TEM-1_ (J01749)	B1	1308
	SHV type	*bla*_SHV-12_^a^ (AY008838)		
	BER type	Five silent mutations (T375C, G378A, C387T, T477G, and T576A) in *bla*_BER_ (EF125541)		
E4A39024	BER type	*bla*_BER_^a^ (EF125541)	B2	648
E4B39025	TEM type	Two silent mutations (C228T and G396T) in *bla*_TEM-1_ (J01749)	B1	224
	BER type	*bla*_BER_^a^ (EF125541)		
E4C44009	CTX-M-1 type	*bla*_CTX-M-3_^a^ (AB976577)	D	648
	BER type	*bla*_BER_^a^ (EF125541)		
E5A41032	TEM type	Two silent mutations (C228T and G396T) in *bla*_TEM-1_ (J01749)	B2	410
	SHV type	*bla*_SHV-12_^a^ (AY008838)		
	BER type	*bla*_BER-v2_ (*bla*_BER_ variant 2) with five silent mutations (T375C, G378A, C387T, T477G, and T576A) and four nucleotide substitutions (G245A, G313A, G469A, and G1012A) in *bla*_BER_ (EF125541), which caused four amino acid substitutions (S82N, A105T, A157T, and G338S) in BER (ABM69263) and was called as BER-v2 (BER variant 2)		
	OXA-1 type	*bla*_OXA-1_^a^ (GU119958)		
E6A43048	TEM type	Two silent mutations (C228T and G396T) in *bla*_TEM-1_ (J01749)	D	648
	BER type	*bla*_BER-v1_ (*bla*_BER_ variant 1) with five silent mutations (T375C, G378A, C387T, T477G, and T576A) and two nucleotide substitutions (G469A and G1012A) in *bla*_BER_ (EF125541), which caused two amino acid substitutions (A157T and G338S) in BER (ABM69263) and was called as BER-v1 (BER variant 1)		
E7A44050DS	CTX-M-1 type	*bla*_CTX-M-3_^a^ (AB976577)	A	10
	IMP type	*bla*_IMP-1_^a^ (AB472901)		
	BER type	*bla*_BER_^a^ (EF125541)		
E7A44050RL	BER type	*bla*_BER_^a^ (EF125541)	A	10
E8A49072	TEM type	Two silent mutations (C228T and G396T) in *bla*_TEM-1_ (J01749)	A	410
	CTX-M-1 type	*bla*_CTX-M-3_^a^ (AB976577)		
	BER type	*bla*_BER_^a^ (EF125541)		
	OXA-1 type	*bla*_OXA-1_^a^ (GU119958)		
E8B49043	TEM type	Two silent mutations (C228T and G396T) in *bla*_TEM-1_ (J01749)	A	410
	CTX-M-1 type	*bla*_CTX-M-3_^a^ (AB976577)		
	BER type	*bla*_BER_^a^ (EF125541)		
	OXA-1 type	*bla*_OXA-1_^a^ (GU119958)		
E9A52022	TEM type	*bla*_TEM-233_^a^ (MH270416)	B1	156
	SHV type	*bla*_SHV-12_^a^ (AY008838)		

^a^ 100% nucleotide sequence identity to each gene described as GenBank accession number.

## Supplementary Materials

The following are available online at https://www.mdpi.com/2076-2615/10/1/28/s1: **Table S1**. Primers used for screening various types of beta-lactamase genes; **Table S2**. Primers for housekeeping genes (*adk*, *fumC*, *gyrB*, *icd*, *mdh*, *purA*, and *recA*) used in MLST analysis; **Table S3**. Primers used for phylogroup determination.
