# Supplementary material for: The Occurrence and Characterization of Extended-Spectrum-Beta-Lactamase-Producing Escherichia coli Isolated from Clinical Diagnostic Specimens of Equine Origin
Source: Animals (Basel). 2019 Dec 21;10(1):28. doi: 10.3390/ani10010028 (PMC7022413; doi:10.3390/ani10010028)
Supplement: Supplementary file 1 [file animals-10-00028-s001.pdf]

animals

The Occurrence and Characterization of Extended-Spectrum-Beta-Lactamase-Producing *Escherichia coli* Isolated from Clinical Diagnostic Specimens of Equine Origin

Leta Elias [1], David C. Gillis [1], Tanya Gurrola-Rodriguez [1], Jeong Ho Jeon [2], Jung Hun Lee [2], Tae Yeong Kim [2], Sang Hee Lee [2], Sarah A. Murray [1], Naomi Ohta [3], Harvey Morgan Scott [1], Jing Wu [4] and Artem S. Rogovskyy [1,*]

Table S1. Primers used for screening various types of beta-lactamase genes.

Primer Name	Sequence (5′ to 3′)	Product Size (bp)	Reference
Bla_SHV-F	CACTCAAGGATGATGTATTGTG	885	[1]
Bla_SHV-R	TTAGCGTTGCCAGTGCTCG	885	[1]
Bla_TEM-F	TCGGGGAAATGTGCGCG	971	[1]
Bla_TEM-R	TGCTTAATCAGTGAGGCACC	971	[1]
Bla_CTM-m_group1-F	AAAAATCACTGCGCCAGTTC	415	[2]
Bla_CTM-m_group1-R	AGCTTATTCATCGCCACGTT	415	[2]
Bla_CTM-m_group2-F	CGACGCTACCCCTGCTATT	552	[2]
Bla_CTM-m_group2-R	CCAGCGTCAGATTTTTCAGG	552	[2]
Bla_CTM-m_group9-F	CAAAGAGAGTGCAACGGATG	205	[2]
Bla_CTM-m_group9-R	ATTGGAAAGCGTTCATCACC	205	[2]
Bla_CTM-m_group8-F	TGATGAGACATCGCGTTAAG	875	[3]
Bla_CTM-m_group8-R	TAACCGTCGGTG ACGATTTT	875	[3]
Bla_CTM-m_group10-F	CCGCGCTACACTTTGTGGC	944	[3]
Bla_CTM-m_group10-R	TTACAAACCGTTGGTGACG	944	[3]

Table **S2.** Primers for housekeeping genes (*adk*, *fumC*, *gyrB*, *icd*, *mdh*, *purA*, and *recA*) used in MLST analysis.

Primer Name	Sequence (5′ to 3′)	Product Size (bp)	Reference
Adenylate kinase-F	ATTCTGCTTGGCGCTCCGGG	583	[4]
Adenylate kinase-R	CCGTCAACTTTCGCGTATTT	583	[4]
Fumarate hydratase-F	TCACAGGTCGCCAGCGCTTC	806	[4]
Fumarate hydratase-R	GTACGCAGCGAAAAAGATTC	806	[4]
DNA gyrase-F	TCGGCGACACGGATGACGGC	911	[4]
DNA gyrase-R	ATCAGGCCTTCACGCGCATC	911	[4]
Isocitrate dehydrogenase-F	ATGGAAAGTAAAGTAGTTGTTCCGGCACA	878	[4]
Isocitrate dehydrogenase-R	GGACGCAGCAGGATCTGTT	878	[4]
Malate dehydrogenase-F	ATGAAAGTCGCAGTCCTCGGCGCTGCTGG CGG	932	[4]
Malate dehydrogenase-R	TTAACGAACTCCTGCCCCAGAGCGATATC TTTCTT	932	[4]

Adenylosuccinate dehydrogenase-F	CGCGCTGATGAAAGAGATGA	478	[4]
Adenylosuccinate dehydrogenase-R	CATACGGTAAGCCACGCAGA	478	[4]
ATP/GTP binding motif-F	ACCTTTGTAGCTGTACCACG	780	[4] *
ATP/GTP binding motif-R	AGCGTGAAGGTAAAACCTGTG	780	[4] *

* Revised primers were obtained from: http://mlst.warwick.ac.uk/mlst/dbs/Ecoli/documents/primersColi_html.

Table S3. Primers used for phylogroup determination.

Primer Name	Sequence (5′ to 3′)	Product Size (bp)	Reference
ChuA.1-F	GACGAACCAACGGTCAGGAT	279	[4]
ChuA.2-R	TGCCGCCAGTACCAAAGACA	279	[4]
YjaA.1-F	TGAAGTGTCAGGAGACGCTG	211	[4]
YjaA.2-R	ATGGAGAATGCGTTCCTCAAC	211	[4]
TspE4C2.1-F	GAGTAATGTCGGGGCATTCA	152	[4]
TspE4C2.2-R	CGCGCCAACAAAGTATTACG	152	[4]

References

1. Pitout, J.D.; Thomson, K.S.; Hanson, N.D.; Ehrhardt, A.F.; Moland, E.S.; Sanders, C.C. Beta-Lactamases responsible for resistance to expanded-spectrum cephalosporins in *Klebsiella pneumoniae*, *Escherichia coli*, and *Proteus mirabilis* isolates recovered in South Africa. *Antimicrob. Agents Chemother*. **1998**, *42*, 1350–4.
2. Woodford, N.; Fagan, E.J.; Ellington, M.J. Multiplex PCR for rapid detection of genes encoding CTX-M extended-spectrum (beta)-lactamases. *J. Antimicrob. Chemother.*, **2006**, *57*, 154–5. doi: 10.1093/jac/dki412.
3. Jouini, A.; Vinue, L.; Slama, K.B.; Saenz, Y.; Klibi, N.; Hammami, S., Boudabous, A.; Torres, C. Characterization of CTX-M and SHV extended-spectrum beta-lactamases and associated resistance genes in *Escherichia coli* strains of food samples in Tunisia. *J. Antimicrob. Chemother.* **2007**, *60*, 1137–41. doi: 10.1093/jac/dkm316.
4. Clermont, O.; Bonacorsi, S.; Bingen, E. Rapid and simple determination of the *Escherichia coli* phylogenetic group. *Appl. Environ. Microbiol.* **2000**, *66*, 4555–8.
